# Inpatient suicide in psychiatric settings: Evaluation of current prevention measures

**DOI:** 10.3389/fpsyt.2022.997974

**Published:** 2022-10-28

**Authors:** Francesca Chammas, Dominique Januel, Noomane Bouaziz

**Affiliations:** Centre de Recherche Clinique, EPS Ville-Evrard, Neuilly-sur-Marne, France

**Keywords:** suicide, secondary prevention, hospitalization, risk factors, anhedonia, environment design, psychotherapy

## Abstract

The risk of suicide in psychiatric hospitals is 50 times higher than in the general population, despite patient safety being a priority for any hospital. However, to date, due to the complexity of assessing suicide risk, there has been no consensus on the suicide prevention measures that should be in place in hospitals. The aim of this work is: To provide an overview of the progress that has been made in the field of inpatient suicide prevention in recent years; discuss the problems that remain; and suggest potential future developments. As new clinical dimensions (notably anhedonia, psychological pain and hopelessness) develop, they should become new therapeutic targets. Team training (like the Gatekeeper Training Program) and the latest advances in suicide risk assessment (such as the Collaborative Assessment and Management of Suicidality) should be implemented in psychiatric wards. Suicide prevention plans (e.g., ASSIP, SAFE-T, etc.) represent easy-to-administer, low-cost interventions. The Mental Health Environment of Care Checklist has been proven effective to reduce suicide risk at hospitals. Furthermore, the types of psychotherapy recommended to reduce suicide risk are cognitive behavioral therapy (CBT) and dialectical behavioral therapy (DBT). There are several pharmacological treatments for suicide risk, such as lithium and clozapine, which have been shown to be effective in the long term, as well as ketamine and esketamine, which are more effective in the short term. Following some encouraging recent results, buprenorphine may also be proposed to patients with a suicide risk. Triple chronotherapy rapidly improves depressive symptoms over 9 weeks. Regarding brain stimulation techniques, rTMS has proven to be effective in alleviating multiple dimensions of suicidality.

## Introduction

According to the World Health Organization, nearly 703,000 people in the world die by suicide each year, at a rate of one death every 40 s (1). Approximately 90% of these individuals suffer from a psychiatric pathology, the most common being mood disorders (2).

Van Orden et al. (3) describe suicidal behavior as a continuum from suicidal ideation to completed suicide. The entire spectrum of suicidal behavior could be seen as a dysfunctional and self-destructive coping strategy (4). Notably, the DSM-5 defines Suicide Behavior Disorder (SBD) as a suicide attempt in the last 24 months. This diagnosis does not apply to suicidal ideation or preparatory acts.

Since the 2000s, the average annual prevalence of suicide by patients hospitalized in psychiatric settings has only been 650/100,000 (5). It is, therefore, a rare phenomenon.

The decision to admit a patient to a psychiatric hospital is largely based on an assessment of the danger he represents to himself or others. Thus, the safety of the patient is an essential prerequisite for admission to a hospital (general or psychiatric). Indeed, the family and friends expect health professionals to avoid risk, and that their loved one will be protected, including from himself (6). However, to date, there is no consensus on the preventive measures to be taken to prevent the occurrence of this event in hospital. This is due to the complexity of assessing suicidal risk. Indeed, if the suicidal risk factors are well known at the collective level, their predictive value is very low at the individual level. So how can we improve the identification of patients at risk? In other words, how can we know who, among the subjects at risk of suicide, will act?

We will first look at the epidemiology of in-hospital suicide, as well as the differences reported according to country, diagnosis and methods used. We will then discuss the components necessary for effective prevention of suicide in hospitals, which include: the latest advances in suicide risk assessment and team training, ways of making hospital wards safe, the types of psychotherapy recommended for this indication, and the treatments to be provided (both those for the underlying psychiatric pathology and those more specific to the suicide crisis).

The aim of this work is to provide an overview of the progress that has been made in the field of inpatient suicide prevention in recent years, discuss the problems that remain, and the future potential developments.

## Materials and methods

English and French language articles published from January 1, 1971 to August 1, 2021 were located on PubMed using the search terms: suicide AND (secondary prevention OR hospitalization OR risk factors OR anhedonia OR psychotherapy OR lithium OR clozapine OR ketamine OR buprenorphine OR transcranial direct current stimulation OR transcranial magnetic stimulation OR electroconvulsive therapy).

We also found some studies via the bibliographies of some papers that were in the results of the PubMed search.

## Epidemiology of inpatient suicide in psychiatric settings

### Incidence and prevalence

Inpatient psychiatric suicide accounts for approximately 5% of all suicides (7–10). The incidence of suicide in psychiatric hospitals is 250 per 100,000 admissions, five times higher than in the general population (11, 12). Ajdacic-Gross et al. (13) found that the risk of suicide in psychiatric hospitals is 50 times higher than in the general population. Foley and Kelly (14) report that the majority of psychiatrists have personal experience of suicide in hospital of at least one of their patients.

According to the meta-analysis by Walsh et al. (5) the number of suicides per 100,000 patient-years has increased over the last three decades, probably reflecting the increased severity of a smaller cohort of patients admitted to psychiatric hospitals in the deinstitutionalization era who are at higher risk of suicide. They find an average prevalence since the 2000s of 650 suicides per 100,000 hospitalized patients.

For Santis et al. (15) suicide in psychiatric hospitals is a rare event, with an estimated prevalence of between 0.1 and 0.4% of all psychiatric admissions.

### By country

According to Combs and Romm (16) the suicide rates of psychiatric inpatients in different countries vary from 100 to 400 per 100,000 admissions.

In France, there is a lack of epidemiological data on suicide in psychiatric hospitals. However, at the Sainte-Anne hospital, between 2009 and 2018, Hauseux et al. (17) found 15 cases of completed suicide among 7,105 hospitalized patients. This would give an incidence of 210 cases per 100,000 hospitalized patients, which is much lower than the current average found in the literature [650 per 100,000 hospitalized patients (5)], but much higher than the average for the general population [15 per 100,000 inhabitants (18)].

In England and Wales, Appleby et al. (19) found that 4% of all suicides were committed by psychiatric inpatients. A report covering the period 2001 to 2011 shows an average of 6,384 suicides in the general population per year (20). Of these, 25% were suicides of “psychiatric patients” (defined as “*patients who had contact with mental health services in the past year*”), within which suicides of psychiatric inpatients accounted for 10%, or 2.5% of all suicides in the general population. Thus, approximately 200 suicides occur each year in psychiatric wards in Britain. Burns et al. (21) report a general population suicide rate in 2016 in the United Kingdom of 13.4/100,000, of which 28% were attributable to patients with mental disorders, of which 9% were attributed to inpatients. Over the period 2005 to 2015 in the UK, 1,443 psychiatric inpatient suicides were recorded (21, 22). According to Sakinofsky (6), the decline in inpatient suicide in the UK is probably an artifact of bed closures and is tempered by a proportional increase in outpatient suicides. Thus the risk of suicide has been transferred from the hospital to the community (23).

In the United States, approximately 1,800 suicides occur in psychiatric units each year (24, 25). Jacobs et al. (26) only estimates 1,500 inpatient psychiatric suicides. In 2008, inpatient suicide was the second most frequent sentinel event (defined as “*an unexpected event involving death or serious physical or psychological injury, or the risk of such an event”*) reported by The Joint Commission (27). There were 16 and 30 hospital inpatient suicides identified in the National Violent Death Reporting System during 2014 and 2015, respectively. It is estimated that between 48.5 and 64.9 hospital inpatient suicides occur per year in the United States, with 31.0–51.7 of these events occurring during psychiatric hospitalization. This is vastly lower than the most widely quoted figure of 1,500 per year, which appears to have been based on speculation (28).

In Malaysia, Siau et al. (29) report that attempted suicide is a crime, which may increase negative perceptions and underestimate the measurement of this event.

In Hong Kong, Dong et al. (30) report an in-hospital suicide rate of 269 per 100,000 psychiatric hospital admissions.

### In psychiatric hospitals

Hauseux et al. (17) emphasize that criteria for hospitalization in psychiatry combines several factors of suicidal risk including a severe mental disorder in the acute phase, a recent suicide attempt, and the effects of stigmatization, especially in the case of hospitalization without consent.

According to Girard (31), between 15 and 60% of admissions to psychiatric institutions are for reasons of high suicidal risk, which explains why the rates of suicidal behavior are higher in this clinical setting, despite the fact that supervision may be more rigorous. Moreover, the mental disorders most associated with suicidal risk are schizophrenia and major depression, which are predominantly found in psychiatric hospitals.

### By diagnosis

Smith and Munich (32) suggest two profiles of patients at high risk of suicide. The first is an acute psychotic state with agitation in a patient with schizophrenia whose evolution is unfavorable. This patient is at risk of committing suicide shortly after admission. The second patient profile is perceived by the team as less seriously ill, presents significant depressive symptoms and is considered to be responsive to treatment. Indeed, Schweizer et al. (33) warn against cases of “hidden bipolarity” where a hypomanic turn could be perceived as an improvement of the suicidal patient. Moreover, antidepressants are initially effective on the physical symptoms of depression (slower movements, lack of energy, fatigue, etc.), and are effective on psychological symptoms (such as suicidal ideations, dark thoughts, etc.) a few weeks later. Some studies (34, 35) have highlighted an increase in suicidality when antidepressant treatment is introduced. Thus, the presence of suicidal ideation during a depressive episode requires an appropriate antidepressant treatment. Suicide attempts can be prevented by informing the patient of the possible increase in suicidal ideation in the first weeks of a new antidepressant treatment, and by scheduling more frequent interviews in order to monitor any exacerbation of the ideation.

In their controlled trial, Sharma et al. (36) analyzed the diagnoses of 44 inpatients in an Ontario psychiatric hospital from 1969 to 1995 who died by suicide. The majority suffered from a mood disorder (25% bipolar disorder, 43% depressive disorder), which is in line with the results of other studies (37–39). The other diagnoses found were: schizoaffective disorder (9%), schizophrenia (14%) and “other” (9%) including anxiety disorder, organic disorder, and substance use disorder.

Numerous studies of inpatient suicide show that schizophrenia and mood disorders are the most common diagnoses of suicide, accounting for nearly 45% (22, 30, 36, 40–45).

Høyer et al. (46) report that psychiatric hospitalization is a period of increased risk for suicide in people with a mood disorder. According to Qin et al. (7), patients hospitalized in psychiatry for a mood disorder are 150 times more likely to die by suicide than people who have never been hospitalized.

In addition, Zeng et al. (47) emphasize the clinical utility of conducting a comprehensive personality assessment when examining suicidal risk in patients with severe mood disorders, as comorbid borderline personality disorder places them at increased risk for suicide.

### By method

The methods vary according to the place of the act. Blain and Donaldson (10) reported that out of 58 suicides of patients between 1987 and 1991, 46% took place inside the hospital, 28% during a discharge, and 26% during a runaway. Thus, the majority of suicides occurred outside the psychiatric institution. This result is found in several studies (48, 49).

Hunt et al. (50) report that in the United Kingdom, hanging was the method for 77% of in-hospital suicides between 1999 and 2007. The most common anchor points were doors, hooks or handles, windows, belts, sheets, or towels. The use of shoelaces, doors, and windows increased over time.

Of the 243 transitions to suicide (178 suicide attempts and 65 completed suicides) that occurred between December 1999 and December 2011 in Veteran Affairs hospitals in the United States, hanging was the method used in 43.6% of cases (and in over 75% of suicide deaths) (25). The other methods were: cutting (21.4%), strangulation (9.4%), and drug overdose (10%). In addition, while belts were used in less than 10% of hangings, they were used as a lanyard in more than 31% of deaths. Similarly, while cabinets and lockers accounted for only 6.6% of the anchor points for hangings, they were used in more than 27% of deaths. This is confirmed in the National Violent Death Reporting System and Sentinel Event databases in 2014 and 2015, in which hanging was the most common method of inpatient suicide, accounting for 70.5% of all inpatient suicide events (28). Of the inpatient hanging events, a door, door handle, or door hinge was the most commonly used fixture point (53.8%). More than 90% of suicides took place in private spaces such as the bathroom, bedroom, closet, and shower.

Ruff et al. (51) analyzed the suicide methods of 436 inpatients in psychiatric hospitals in Switzerland between 2000 and 2010. Two-thirds of these suicides took place outside the hospital, with the most common methods being: Jumping under a train, jumping from a height, and drowning. Inside the unit, the most used methods were hanging, self-defenestration, and voluntary drug intoxication.

## Prevention of inpatient suicide in psychiatric settings

The safety of the patient is a prerequisite for admission to the hospital, and the patient’s entourage expressly expects the team to protect them, including from self-harm (6).

### Suicide risk assessment

#### Limitations of our current practices

According to Wingate et al. (52), the absence of reliable tools for recognizing suicidal risk results in the clinician *either* underestimating the danger of the situation *or* overestimating it, in which case he or she uses excessive caution that may deprive the patient of certain fundamental rights. This lack of reliability in suicide risk assessments causes divergence in results between professionals for the same patient (53).

In their study of 76 inpatient suicides, Busch and Jacobs (54) showed that suicide risk assessment and standard precautions are ineffective: 77% of patients denied having suicidal thoughts at their last interview and 42% were monitored every 15 min. Meehan et al. (55) reported in their study that 72% of patients received a minimal level of monitoring at the time of death because the team judged them to be at low or no risk for suicide.

Paris (56) states that over-caution is the result of a “law of fear” that leads many patients to be hospitalized because of the clinician’s fear that they will commit suicide, not because the clinician believes that hospitalization is the best possible management. He adds that although many patients benefit from the protective and containing environment of the hospital during a suicidal crisis, hospitalization of chronically suicidal patients is counterproductive and should most often be replaced by outpatient management. For example, an intensification of the suicidal risk resulting from hospitalization has been identified particularly in patients with borderline personality disorder (57).

According to Ryan and Oquendo (58), “*diagnostic expertise and knowledge of risk factors and warning signs of suicide (guided clinical assessment), combined with judgment derived from clinical experience, remains the clinician’s quintessential suicide risk assessment.”* Clinician intuition alone is considered unprofessional. For the assessment of suicidal ideation, the Columbia Scale appears to be the most valid instrument.

So, what are the risk factors for suicide?

Jacobs et al. (26) found common characteristics of patients whose suicides were completed during hospitalization: young age, male gender, suicide attempt as a reason for hospitalization, family history of suicide, personal history of severe psychiatric disorder with previous hospitalizations. It should be noted that these non-modifiable risk factors offer little room for action by clinicians.

For Busch and Jacobs (54), severe agitation and a very high degree of anxiety (present in 79% of the 76 patients who committed suicide) are better predictors of completed suicides. Bowers et al. (22) reach the same conclusion.

De Leo and Sveticic (59) divide risk factors into two categories: individual factors (including history of suicide attempts, chronic psychiatric disorder especially thymic and psychotic disorders, presence of hopelessness, recent stressful life events especially bereavement, psychiatric or somatic comorbidities, alcohol or drug abuse, family history of suicide, male gender, unemployment, conflicting family environment) and factors related to treatment (including admission for suicide attempt, long hospital stay, non-compliance, multiple hospitalizations, numerous therapeutic changes, highly fluctuating clinical picture, removal of inhibition secondary to antidepressants). However, although all these risk factors have a strong statistical association with suicide, their sensitivity is insufficient to accurately predict the probability of such an event. This is why other studies have focused on dynamic risk factors whose presence would acutely indicate an increased risk of suicide: anxiety and anhedonia (60), non-adherence, social isolation, very strong awareness and insight into the pathology and its social consequences (41).

Qin and Nordentoft (61) also found that the first week of hospitalization and discharge were the most critical periods. Hauseux et al. (17) warn that caregivers’ vigilance decreases during hospitalization, as their attention is focused on new admissions.

Santarelli (62) explains that the identification of risk factors in a given patient is poor for predicting the occurrence of suicide because of low sensitivities and specificities. Klein (63) argues that even when a patient has multiple risk factors, which is common in hospitalized patients, the rarity of a suicide results in low predictive value and therefore too many false positives and false negatives. Large et al. (64) state that despite the strong association between the “high risk” category and completed suicide, the low incidence of in-hospital suicides implies that the positive predictive value of the “high risk” category is 1.4%, i.e., out of 100 patients categorized as “high risk for suicide,” more than 98 will not commit suicide. They suggest that the development of safer hospital structures and environments, and improved systems of care, are more likely to reduce suicides than an individual patient assessment of suicidal risk.

Santarelli (62) explains that risk factors are used to identify groups of patients with an increased likelihood of suicide in the long term, but they are only very randomly used to predict a short-term (hours, days or weeks) suicide. Thus, during the assessment of a patient, risk factors are reviewed, but this approach only works on a group level, not on an individual level. It gives visibility on the patient’s vulnerability over the next few years, but in the immediate future does not inform us about the degree of urgency of the situation.

Substance use disorder (SUD) is considered an important risk factor for suicide, with vulnerable categories identified as having a younger age, a history of psychiatric care, and consuming opiates or alcohol (65). Indeed, substance use, substance intoxication, and pathological substance use have been demonstrated to be positively associated with suicidal behavior (65). Drug misuse or chronic drug abuse may impair judgment, weaken impulse control, and/or interrupt the functioning of critical inhibitory neurotransmitter pathways, thereby leading to enhanced suicidal tendencies driven by disinhibition (66). Evidence indicates that, compared to the general population, individuals who consume alcohol or other drugs have a 10–14 times greater risk of death by suicide (66). In their study, Martinotti et al. (66) found high scores of suicidality in users of psychodepressors, specifically opioids, among a sample of young adults who had a high level of education and a good socioeconomic status. The authors add that high levels of suicidality did not specifically correlate with undergoing psychopathological conditions, thereby indicating an independent association between the use of psychoactive substances and suicidality. Chiappini et al. (65) stressed that early interventions in suicide prevention should include the identification of potential risk factors, such as SUD, as well as the abuse of licit/illicit drugs and new psychoactive substances, which must be explored, assessed, and addressed in the management plan of likely suicidal thoughts or behaviors.

Another risk factor to have in mind is alexithymia, which is characterized by difficulties in identifying and verbalizing feelings, a paucity of fantasy life, concrete speech and thought closely tied to external events (67). The authors add that alexithymic individuals also suffer from affective dysregulation, the inability to self-soothe and manage emotions because of a lack of emotional awareness. The presence of alexithymic traits in individuals with major depression may be related to higher disorder severity and higher suicide risk (68). Several studies have demonstrated that alexithymic subjects with several psychiatric disorders may have a higher disorder severity and an increased suicide ideation than non-alexithymic subjects (69). In their study, De Berardis et al. (70) found that when considering the dimensions of the alexithymia construct, the Difficulty of Identifying Feelings dimensions (measured in the TAS-20) were found to be significantly associated with suicide ideation, and this finding confirms previous studies. De Berardis et al. (70) add that alexithymia screenings and follow-up care seem to be useful for suicide ideation screening and prevention. In the case of positivity for alexithymia, emotions-specific preventive intervention strategies for such subjects should be provided to directly reduce alexithymia severity and indirectly improve resilience and suicide ideation. Moreover, De Berardis et al. (70) sought to know if level of homocystein, an neurotoxic amino acid (71), levels in PTSD patients would depend on concurrent presence of alexithymia or not. They didn’t find any relationship between alexithymia an levels of homocystein. Recently, a study evaluated 14 potential biomarkers of suicidal risk and found that cortisol, cholesterol and folate levels predict suicide attempts; also homocysteine and interleukin 1-beta predict suicidal severity (72).

Although there have been some attempts to develop algorithms that are sensitive enough to predict suicide risk (40, 49), the very small number of completed suicides precludes the reliability of these models (73). Prevention relies on better prediction. Therefore, there is an interest in exploring new transnosographic dimensions which can be easily measured and which may be used as therapeutic targets (74).

#### Clinical dimensional approach

The challenge is to determine who, among the subjects at risk of suicide, will act. New clinical dimensions have been studied to help us answer this question.

##### Anhedonia

The first clinical dimension is anhedonia, defined by Treadway and Zald (75) as reduced interest and inability to experience pleasure. According to a meta-analysis that grouped together 15 studies (approximately 7,000 patients), anhedonia was found to be more important in subjects with suicidal ideation than in subjects without such ideation, independently of comorbidities or level of depression (76). Anhedonia is a predictive factor of suicidal ideation (77–79), suicide attempt (80), and completed suicides (81, 82) up to 1 year of follow-up.

Ducasse et al. (83) evaluated the link between anhedonia and suicidal events (ideation and attempts) in 2,839 subjects suffering from mood disorders, followed for 3 years. They measured anhedonia with the French version of the Snaith-Hamilton Pleasure Scale. During the follow-up, people complaining of anhedonia were found to be at greater risk of suicidal ideation but not of acting on it, adjusting for confounding factors (treatment, history of suicide attempt, severity of depression, etc.). However, they point out that their results could suggest a 25% increase in suicidal risk in anhedonic patients during follow-up. According to them, if depression explains the association between anhedonia and suicide attempt but not between anhedonia and suicidal ideation, we can hypothesize that suicidal ideation acts as an avoidance strategy of a temporary state of anhedonia, whereas a suicide attempt occurs when anhedonia persists because it is related to depression.

According to Ducasse et al. (83), the prevention of suicidal risk must target the two components of “hedonia”: That in which one “desires” something (called motivational hedonia) and that in which one likes something, or appreciates an experience (called consumption hedonia). This would provide a better understanding of suicidal pathophysiology.

Motivational hedonia is thought to be linked to the dopaminergic system. Dopaminergic neurons in the ventral tegmental area join the mesolimbic pathway, which is associated with motivation and reinforcement learning (84). This connection is essential for the function of the ventral and dorsal striatum (85), two regions associated with motivation and reward anticipation (86, 87). Thus, anhedonia and suicidal behaviors could result from a decrease in dopamine release in the striatal circuit (88) which would lead to an alteration of the reward circuit and decision making (89, 90). Conversely, high levels of dopamine in the ventral striatum have been positively correlated with motivational hedonia (or reward anticipation) (91), but not with consumption hedonia (92). Thus, dopamine levels in the striatum are strongly correlated with motivation and effort, and any abnormality in the dopamine circuit could lead to alterations in reward-seeking behavior (93).

Consumption hedonia, the pleasure derived from an experience, is not directly linked to dopamine (94), but to the opioid system. Microinjections of opioids into the ventral striatum (95) and the ventral pallidum (96) increase the hedonic responses of consumption. Opioids modulate dopaminergic transmission and are involved not only in consumption hedonia (97) but also in pain regulation (98).

##### Psychological pain

The second dimension highlighted is psychological pain. A meta-analysis of 20 studies shows that the level of psychological pain is higher in subjects with a history of suicide (recent or past) and in subjects with current or past suicidal ideation, compared with controls, and this is always independent of the level of depression (99).

In their study, Alacreu-Crespo et al. (100) surveyed 372 depressed hospitalized patients regarding the level of psychological pain. They collected information showing the presence of a suicidal event (defined as hospitalization for suicidal ideation, suicide or completed suicide) within 1 year after the survey. They found that the maximum level of pain over the last 15 days was predictive of the occurrence of a suicidal event at 1 year, independent of depression, history of suicidal ideation and treatments received. It would therefore be relevant to ask patients to estimate on a numerical scale the level of current psychological pain but also maximum pain over the last 15 days.

It should be noted that psychological pain and suicidal behavior are often precipitated by interpersonal difficulties in everyday life. Based on this observation, Olié et al. (101) were interested in predicting the occurrence of psychological pain in the natural environment using functional MRI data. They subjected 33 euthymic patients with a history of depression, with or without past suicide attempt, to a social exclusion test during functional MRI. During a “cyber ball game,” each patient played and interacted in a computerized virtual reality with other characters. The patients were included in the first phase of the game, but then did not receive the ball anymore. After the test in the MRI, the patients had to report their psychological pain level five times a day for 1 week using the EMA (Ecologic Momentary Assessment) scale, specifying the context in which they were at the time of the recorded emotional state. The MRI analysis focused on regions of interest known to be involved in suicidal vulnerability (prefrontal, anterior cingulate and insular regions) to correlate experimental exclusion with the occurrence of psychological pain in ecological situations. They found a negative correlation between daily ratings of psychological pain and orbitofrontal activation for exclusion vs. inclusion during the cyber ball game in subjects with prior suicide attempt, but not in controls. Note that dysfunction of this orbitofrontal cortex underlies the impaired decision making found in suicidal subjects, even in the euthymic phase. It can be hypothesized that people vulnerable to suicide, who experience psychological pain in their daily lives, would be less inclined to activate this orbitofrontal cortex in a social context. This could lead to disadvantageous decision making and impulsive choices that could contribute to suicidal acts.

Furthermore, the risk factor “hopelessness,” which can be considered as a factor of psychological pain, is defined as a dimension characterized by negative expectancies for the future, lack of general motivation, and the attribution of wrong interpretations to personal experiences (102). From a clinical point of view, a lack of positive expectations for the future combined with the distress that often occurs during a major depressive disorder leads to an increased risk of suicide, because it appears to be the last and only solution to their inner “unsolvable problems.” In fact, hopelessness seems to be more closely related to suicidal ideation than suicidality is related to depression severity. In addition, hopelessness could be considered as a clinical predictor for any suicide attempt, even when depressive symptoms are contained (102). Notably, in their study, Pettorrusso et al. (102) also found a relationship between hopelessness in major depressive disorder and striatal dopaminergic dysfunction.

##### Endophenotypes

Gould et al. (103) propose a number of stable clinical dimensions (“endophenotypes”) that could be a gateway to the development of new therapeutic strategies. They plan to study the neurobiology of suicide through animal modeling. This would make it possible to test new hypotheses, first in animals and then in humans, and to pave the way for new treatments for suicide prevention. Of course, suicide as it exists in humans is the result of a unique cognitive process that is not found in animals. Thus, animal models are only thought to represent certain aspects of human pathologies, or certain neurobiological components involved in psychiatric disorders. This approach makes it possible to circumvent the obstacles inherent in studies on humans (ethical issues, numerous comorbidities and confounding factors, insufficient number of subjects, etc.). Endophenotypes such as aggressiveness, impulsivity, and maladaptive decision-making processes have been associated with suicidal behaviors and represent new therapeutic targets to be explored.

Those three clinical dimensions (anhedonia, psychological pain, endophenotypes) are summarized in Table 1.

**TABLE 1:** Clinical dimensions.

Clinical dimensions	Predictive factor of…	Can be measured by…	Pathophysiology
Anhedonia (76)	Suicidal ideation, suicide attempt, completed suicide Up to 1 year of follow-up	Snaith-Hamilton Pleasure Scale	2 Targets: - Motivational hedonia (desire): dopaminergic system - Consumption hedonia (pleasure): opioid system
Psychological pain (99)	Maximum level of pain over the last 15 days: predictive of the occurrence of a suicidal event at 1 year	Ecologic Momentary Assessment Scale	Hypoactivation of orbitofrontal cortex in social context in suicidal subjects ⇒ impaired decision making and impulsive choices
Endophenotypes (aggressiveness, impulsivity, maladaptive decision-making) (103)	New therapeutic targets to explore using animal modeling		

#### Current value of biological predictors

In the case of neuroimaging and for the identification of biological markers of suicide risk, the way forward is the identification of neural signatures. A neural signature is defined as a set of brain activation features that are based on the decomposition of certain concepts into relevant compounds using imaging and machine-learning techniques. Just et al. (104) give the concept of “spoon” as an example, which is based on elements such as manipulation (localized in motor regions) or eating and tasting (localized in the insula and inferior frontal gyrus). These signatures would allow the identification of brain patterns capable of predicting mental outcomes and behaviors at the individual level. These signatures, which are biological markers of altered conceptual representation, could complement and improve the accuracy of clinical assessment of suicidal risk.

The study by Just et al. (105) focuses on a biological measure that would assess alterations in neural representations of concepts related to life and death in individuals with suicidal ideation. This approach is based on recent advances in cognitive neuroscience that use machine-learning techniques to identify a concept from its signature in functional MRI (104, 106, 107). Using this approach, Just et al. (105) identified 17 subjects with suicidal ideation and 17 controls with 91% accuracy, based on their functional MRI results. A similar classification made it possible to distinguish, with 94% accuracy, nine subjects with suicidal ideation who had already made a suicide attempt from eight subjects with suicidal ideation and no history of suicide. Moreover, an important aspect of these conceptual alterations is the emotion evoked in the participants, whose neural signature was used as an alternative basis for a classification with 85% accuracy. This study therefore proposes a biological and neurocognitive basis for highly accurate classification of participants with suicidal ideation through recognition of their altered conceptual representations. This would also allow the recognition of suicidal patients who conceal their decisions and suicidal scenarios from the clinician. In fact, nearly 80% of patients who die by suicide deny having suicidal thoughts during their last contact with a health professional (54).

Just et al. (105) emphasize that the neural signature is a biological marker that can be broken down into several thought processes. This allows for the precise identification of the element that has been altered, namely, the emotional component. Indeed, subjects with a history of suicide attempts represent certain concepts (related to suicide) differently than the general population (108, 109). Based on an archive of neural signatures identified in neurotypical subjects, two studies (105, 106) looked for the presence of four emotions that have been detected in suicidal patients (110–112): sadness, shame, anger and pride. Just et al. (105) hypothesized that the degree of presence of these emotions in the neural signatures of concepts (e.g., “death”) would allow categorization of patients. The concepts that allowed for most discrimination between patients with suicidal ideation vs. the control group were “death,” “cruelty,” “disorder,” “carelessness,” “good,” and “praise.” For the brain, regions that allowed for the most discrimination included the left superior fronto-medial area, frontal/anterior medial cingulate, right middle temporal area, left inferior parietal area, and left inferior frontal area. In patients with suicidal ideation compared to the control group, the concept of “death” evoked more shame, the concept of “disorder” evoked more sadness and less anger, and “recklessness” evoked less pride. The concepts that best discriminated between those who acted out and those who did not were: “Dead,” “inanimate,” “reckless” and the regions of interest were the left superior fronto-medial area, the medial frontal/anterior cingulate, and the right middle temporal area. The concept of “death” evoked less sadness in suicidal patients who had previously experienced a suicide attempt than in suicidal patients who had never experienced a suicide attempt. Suicidal patients as a whole felt more ashamed when the concept of “death” was evoked than the control group. Thus, the emotional component of the altered concepts in suicidal patients could be a target to consider in psychotherapy. Moreover, the brain regions involved in the representation of neural signatures could also be therapeutic targets for brain stimulation techniques (tDCS, TMS). Just et al. (105) specify that one avenue to be explored is the capacity of this neurosemantic approach to predict imminent suicidal risk. This would require a longitudinal study with a larger cohort.

The concept of neural signatures is summarized in Table 2.

**TABLE 2:** Neural signatures.

	Neural signatures
Definition (104)	Biological markers of altered conceptual representation using functional MRI and machine-learning
Makes it possible to distinguish …(105)	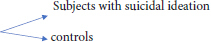
	subjects with suicidal ideation 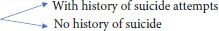
Thanks to … (105, 106)	The emotional component: a potential target for psychotherapy or brain stimulation techniques ex: For patients with suicidal ideation vs. control group, the concept of “death” evoked more shame, “disorder” → more sadness and less anger, “recklessness” → less pride.
Future prospects (105)	Recognition of suicidal patients who conceal their decisions

### Team training

#### Questioned practices

##### Anti-suicide contracts

A practice still widely used by nurses on psychiatric wards is to make “anti-suicide contracts” with patients. There is little evidence in the literature that this is effective and, indeed, there is no standardized technique for implementing this measure. This may increase the risk of fatal error (113). An anti-suicide contract is a verbal or written agreement with the patient. The patient agrees not to engage in suicidal ideation, self-injury, and/or to seek help when suicidal ideation reaches too high a level, in this case, increased treatment may be necessary (114). According to Puskar and Urda (115), anti-suicide contracts are used in 79% of Ohio psychiatric units. Nurses are the primary negotiators of these contracts (116). Indeed, as the nursing team is in contact with patients 24 h a day, it is their responsibility to maintain a safe environment. Introduced by Drye et al. (117), the aim of this measure was to involve patients in their suicidal assessment by letting them determine whether or not they were going to commit suicide. Of the 600 cases examined, the authors reported a 0% death rate from suicide for patients who had signed anti-suicide contracts. At the time, this generated a lot of attention, however, since then, many articles have found that the evidence for the use of anti-suicide contracts is lacking or non-existent. For example, 41% of 152 psychiatrists surveyed, had patients who committed suicide or severe suicide attempt after entering into an anti-suicide contract (118). Drew (119) reported that 65% of patients who made these contracts subsequently committed self-harm on the unit, and that in a review of 650 patients over a 6-month period, there was no evidence of the effectiveness of anti-suicide contracts in preventing self-harm. Farrow (120) describes that anti-suicide contracts can even be dangerous in community crisis situations. In such situations, the nurse does not have time to develop a therapeutic alliance and mutual trust with the patient, so the patient’s agreement to an anti-suicide contract may be a response to feeling pressured by the nurse rather than a voluntary agreement. In addition, “strong opposition” to this technique on the part of patients and nurses has been reported (121). Patients expressed a lack of confidence in their ability to enter into a contract when they are already in an altered cognitive state due to the acuteness of their psychiatric disorder. Several added that they simply viewed the contract as a way to prolong or terminate a hospitalization. Some nurses in the study also admitted to using the contract for their own legal protection rather than in the best interests of the patient. Indeed, the fundamental element for this technique is the existence of a therapeutic alliance. This may be the reason for the ineffectiveness of anti-suicide contracts used today in services where the length of hospitalization can be reduced to a few days, leaving no time for the nurse to build a relationship of trust with the patient. The patient will not feel comfortable honestly revealing his or her intentions to commit suicide. In turn, the nurse, who does not know the patient well enough, may miss subtle cues that indicate the patient’s ambivalence to enter into the contract. In addition, the term “contract” should be avoided, as it can lead to an additional burden on the patient, and a false sense of security for the clinician about the risk of acting out (121). Farrow (122) also mentions that in her study, some nurses were making suicide contracts to relieve their own anxiety about patient safety.

An important aspect to consider is the legal concept of “predictability”: Clinicians are expected to predict a patient’s suicidal behavior (123). It should be noted that anti-suicide contracts are not sufficient to protect against legal liability (124). Indeed, evaluators sometimes make incomplete assessments of suicidal risk after having given the patient an anti-suicide contract.

Thus, relying solely on an anti-suicide contract as a tool for assessing suicidal risk reflects poor clinical judgment and practice, as well as inadequate legal protection (124).

##### Formal observation

Formal observation is a monitoring protocol used by psychiatric nurses that includes routine monitoring, 15–30 min monitors, constant monitoring, and one-to-one monitoring (125).

Sakinofsky (6) reports that traditionally, formal observation of suicidal patients was considered an intrinsic task of the psychiatric nursing profession. However, over the past 20 years or so, this practice has become controversial. Nurses argue that it has not proven to be effective (125). They feel it is intrusive, humiliating for patients, and goes against their humanistic values and any therapeutic alliance (126). On the contrary, some patients interviewed (127–129) consider that although the experience is intrusive, they all feel safer and more hopeful when the observers are optimistic, provide emotional support and interact therapeutically with them.

According to the Ontario Good Nursing Practice Recommendations (130), this is consistent with the empirical finding that maximum and constant observation beyond 72 h can become counterproductive (131). These recommendations recall the definitions of the 4 levels of observation (132):

• Level 1: General observation. It is not necessary to keep all patients within sight, but their location should be known to staff at all times.
• Level 2: Intermittent observation. The patient’s location should be monitored every 15 min. The recommendations of Lieberman et al. (133) believe that at this level it is best to perform checks at varying intervals of less than 15 min so that the patient cannot predict the exact time of the next check.
• Level 3: Within sight. There is a high risk of a self- or hetero-aggressive act. Therefore, the team must be prepared for this eventuality 24 h a day, although it is clear that observers may not be close enough to intervene effectively.
• Level 4: Within reach. Highest risk level that requires the patient to be “within reach” of a nurse at all times, including the bathroom.

Note that in the case of an unpredictable, impulsive, or aggressive psychotic patient, several nurses may be assigned to observation at the same time. On the other hand, level 4 unsurprisingly requires a very large number of caregivers, is very expensive to fund, and is stressful for nurses. For this reason, some hospitals have developed acute care units where multiple patients can be housed in transparent cubicles and monitored from the nursing station. Closed circuit cameras can be used, but a suicidal patient can easily find the blind spot. The design of future hospitals will likely allow a suicidal patient to be monitored at any time, if necessary (6).

#### Promising new leads

It is the interaction of different training and skills that will exponentially increase the chances of saving lives (15). Santarelli (62) proposes that in the event of a suicidal crisis on the ward, a meeting should be held with the whole team to inform them of the means implemented to prevent the act, and emphasize the fact that in the unit, everyone staff member involved. On this point, Lieberman et al. (133) caution that maintenance staff should be trained to ensure that their products are kept safe and that their carts are always under supervision. Visitors should also be instructed not to bring dangerous items such as plastic bags.

Suicide prevention training for nurses is often too short or outdated. Although mental health care providers are expected to have expertise in the management of suicidal patients, this is a relatively neglected area in the training of mental health professionals (134).

At the Veterans Affairs Hospital in the United States, suicide prevention training is a priority. All hospital staff are required to attend a 1-h general suicide prevention training session each year, provided by the suicide prevention coordinator or another member of the prevention team (135). All physicians have additional on-line clinical training. All new employees must complete the Gatekeeper Training program. Team members responsible for facility security are also required to complete risk reduction training. In keeping with this hospital’s strategy of implementing only evidence-based practices for suicide prevention, all mental health professionals have been trained in CAMS (Collaborative Assessment and Management of Suicidality) (136). The CAMS is a systematic assessment, intervention, and monitoring tool for suicidality recommended for the veteran population in systematic reviews (137–139). The objective of these trainings is to acquire skills such as: systematic risk assessment, recognition of a suicidal crisis, knowledge of monitoring protocols, interventions to decrease suicidality, therapeutic communication specific to suicide, and communication between professionals (60, 140). Santis et al. (15) report that the implementation of all these measures since 2007 in this unit of the Veterans Hospital has been effective. Indeed, from 2003 to 2007, there were a total of five suicide attempts and zero suicides, whereas from July 2007 to 2014, there were no suicides or suicide attempt on the unit.

At present, as we have no variable to predict suicidal risk, the systematic use of certain scales allows us not to miss an indication of suicidality, or even to “unmask” a suicidal project hidden by the patient. Scales such as The Non-Suicidal Self-Injury Assessment Tool (NSSI-AT), which is still able to unmask real suicidal intentionality (141), the Minnesota Multiphasic Personality Inventory Scale (142) for detecting borderline disorder most associated with suicide risk; the Beck Scale for Suicide Ideation (SSI) (143); the Suicidal Affect-Behavior-Cognition Scale (SABCS) (144).

The Suicide Assessment Five-step Evaluation and Triage (SAFE-T) tool (145) incorporates the American Psychiatric Association’s practice guidelines for suicide assessment. The first step is identifying suicide risk factors and note those that can be modified to reduce the risk. The second step consists in noting the protective factors, particularly those that can be enhanced. The third step is to identify suicide thoughts, plan, behavior and intent. The fourth step is to assess the risk level and choose the appropriate intervention to address it. The final step is to determine a treatment plan (medication, psychotherapy, setting…).

At the Grenoble University Hospital, a “high suicide risk patient guide” has been put in place. In agreement with this, Santarelli (62) finds it desirable for patients identified as being at high risk of committing suicide to rely on a document that promotes good coordination of care adapted to each situation.

### Securing the environment

De Leo and Sveticic (59) consider that the premises of a psychiatric unit plays a major therapeutic role; it must induce stabilizing and comforting effects and the architecture must be suicide-proof. Several recommendations have been made in this regard, such as securing open spaces to prevent runaways, removing all sharp or hanging features, and ensuring that units are away from tall buildings or busy roads (133, 146).

According to Sakinofsky (6) an ideal psychiatric unit would be one that allows direct vision of all patients at all times and keeps them safe from physical harm. The premises of the units are implicated in 84% of suicides, which makes them the most important risk factor for in-hospital suicide (133). Benensohn and Resnik (147) asked their patients about structural weaknesses on the unit that could be exploited for suicide. As it turned out, the majority of patients had already explored the potential for a suicide attempt on the unit and were delighted to reveal many points of vulnerability that the health professionals had not thought of.

Other programs have focused more directly on space modifications to decrease the risk of suicide on psychiatric units (148). These efforts have been based on practical recommendations derived from shared clinical experience (149), the analysis of potential hazards using industrial engineering techniques (150), and finally, from December 1999 to June 2006, the analysis of the causes of 52% of the suicides that occurred in veterans’ hospitals (135). This work lead to the development of the Mental Health Environment of Care Checklist (MHEOCC), which was first tested in 113 VA hospitals, then mandatorily implemented in 2007 in all VA hospitals. It led to a reduction of 8,298 risk items in VA mental health units during its first 2 years of use, as well as a reduction in suicide rates from 2.64/100,000 admissions to 0.87/100,000 (*P* < 0.001) (148). Note that during this same period, the suicide rate in US non-veteran hospitals decreased from 45 to 28/100,000 admissions. (25). Furthermore, the decrease in suicides has been maintained over 7 years with a consistent downward trend since the implementation of the MHEOCC (151).

Mills et al. (152) analyzed the causes of 185 suicides and suicide attempts done in VA hospitals. They found that doors and cabinets accounted for 41% of the anchor points for hangings. Therefore, they recommended in the MHEOCC the elimination of all unnecessary doors, removed doors from cabinets, and replaced rods and hangers with shelves. However, removing doors raises other complexities: some doors are fireproof, those in bathrooms are required for privacy (135). So, they collaborated with interior designers to imagine as many alternatives as possible to replace doors (for example, walking around a wall can replace the need for a door). Where doors were to remain, they designed alarm doors and anti-ligature door handles.

Mills et al. (135) also found that objects made into weapons accounted for 14% of identified hazards. Drawers, moldings, cords, tiles, flatware, chairs, artwork, and virtually any small object can be used to harm others or oneself and should be carefully examined. Yeager et al. (153) also advised looking for heavy panels on furniture or heaters that could be removed and used as a weapon or to break windows, as well as specific protocols to ensure that dangerous objects entering the unit (cleaning products, etc.) and objects already in the unit (meal trays, cutlery.) are not left unattended.

It is strongly recommended that regular tours of the facility be conducted to identify potential hazards (152). These are done twice per year in the Veterans Affairs hospitals (19). These rounds are led by the suicide prevention coordinator and the patient safety manager (a nurse) and include the nurse manager, unit manager, engineers, and mental health providers (15). The protocol is to complete the MHEOCC, Risk Assessment and Tracking Form, and establish a corrective action plan. The most common hazard identified was ligature points capable of supporting the weight of a person weighing over 45 kg (135). Gunnell et al. (154) report that 50% of suicides by hanging have a ligature point less than the height of a person. It is therefore important to identify all of them, whether they are high up or even very close to the floor, especially in bedrooms and bathrooms, which are at higher risk of the act due to isolation. In fact, Mills et al. (25) even propose a decision tree for optimizing controls over potential hazards in the facility, in which they suggest that simulation of a suicidal act can guide action regarding mitigation or elimination of potential hazards. Such simulation could include measuring the weight an anchor point can support, or measuring the time it would take a patient to fashion a rope. This framework could encourage staff to invest more time and resources in reducing the most dangerous hazards.

### Psychotherapy in the hospital

Although drug therapy is an important aspect of suicide prevention, suicide cannot always be prevented despite optimal dosage adjustment (155). Suicide prevention in psychiatric hospitals also requires psychotherapeutic interventions that should be systematic, which would also allow for better patient compliance.

Results from randomized controlled trials provide evidence for the effectiveness of cognitive behavioral therapy (CBT) and dialectical behavioral therapy (DBT), particularly problem-solving strategy training (156–158). However, evidence supporting the value of these therapies and how to apply them is still scarce, and further research is needed to confirm the findings and develop treatment plans to employ the best possible therapeutic approach for suicidal patients in a variety of settings including the emergency department, outpatient unit, and inpatient unit (159).

In their review of observational studies, Méndez-Bustos et al. (160) also found that CBT and DBT appear to be the most widely used and effective psychotherapies for patients with suicidal ideation or a history of suicidal ideation, with even rapid efficacy. The authors nevertheless point out that CBT and DBT should not be the only options in the range of possible psychotherapeutic interventions in suicide prevention. For example, the following represent promising avenues for consideration: mindfulness strategies, integrative programs, STEPPS (Systems Training for Emotional Predictability and Problem Solving), and PS-CCI (Problem-solving and comprehensive contact intervention) (160).

#### Dialectical behavioral therapy

DeCou et al. (161) explain that the term “dialectic” here expresses both the multiple tensions that can arise between the therapist and suicidal and borderline personality disorder patients, as well as the emphasis on improving thinking patterns to replace rigid, dichotomous thinking. DBT is based on the theory that the problem to be treated is an ongoing dysregulation of emotions that leads to impulsive and maladaptive behaviors such as self-directed violence, as well as the inability to be flexible in responding to life events. Suicidal events are one of the priority therapeutic targets of DBT.

There are five treatment strategies in DBT:

a) Dialectical strategy
b) “Basic” strategy (validation and problem solving), including standard CBT procedures (behavioral assessment, psychoeducation, contingency management, exposure, etc.)
c) Communication strategies
d) Case-management strategies (environmental interventions)
e) Structural strategies (defined objective of a session)

The therapist must find a “balance” between all of these strategies, to maintain therapeutic progress in a patient who will constantly oscillate between suicidal outbursts, rigid refusal to collaborate, rapid emotional escalation, and real engagement in psychotherapeutic work. The strategies listed can be applied in four different modalities: individual psychotherapy, group psychotherapy, out-of-session coaching, and therapist team meetings. Individual sessions are organized according to a hierarchy of therapeutic targets, the priority being life-threatening behaviors, self-directed violence, and violence toward others.

DBT decreases suicidality in chronic inpatients, however, its implementation is often limited by the patient’s length of stay (162). DBT therapy usually lasts for 1 year (161). It is conceivable that DBT could at least be initiated during hospitalization, as has been done with promising results in an adolescent unit (163).

#### Cognitive behavioral therapy

In their meta-analysis, Hawton et al. (164) show that CBT has superior results to other forms of psychotherapy in the repetition of self-injurious acts, with a follow-up of up to 12 months, which is consistent with the results of another study (165). A 1-week internet-based CBT program (166, 167) has been shown to be effective in reducing suicidal ideation. CBT applied to suicide risk reduction can also be adapted to inpatient groups (168).

Brown et al. (169) in their randomized controlled trial, evaluated a Cognitive Therapy Specific to Suicide Prevention (CT-SP) intervention. They compared two groups of 60 patients—outpatients and inpatients—who had committed suicide in the last 48 h (a “CT-SP plus usual care” group and a “simple usual care” group). The objective was to measure the effectiveness of CT-SP in reducing the frequency of recurrent suicide attempts during an 18-month follow-up. Participants received 10 sessions of CT-SP. The central feature of this psychotherapy was to identify the core thoughts, images, and beliefs activated just prior to the suicide attempt, and to help patients develop adaptive strategies in the face of stressors. The main vulnerability factors that were addressed included hopelessness, poor problem-solving skills, impaired impulse control, non-compliance, and social isolation. Toward the end of therapy, a relapse prevention task was assigned to the patient: they had to reactivate, in session, the thoughts, images, and feelings associated with their previous suicide attempt and determine whether they were able to respond to them with a better adaptive strategy. Successful completion of this task warranted completion of the treatment. If not, additional sessions were planned. The results of this study showed that participants in the CT-SP group were 50% less likely to have a suicide attempt during follow-up than those in the control group. Patients in the CT-SP group also expressed significantly less hopelessness than the control group at 6 months, which encourages the practice of CT-SP. Indeed, Dahlsgaard et al. (170) reported that participants whose level of hopelessness did not change over the course of psychiatric treatment, were more likely to commit suicide. In addition, M. A. Young et al. (171) noted that stable levels of hopelessness in individuals in remission from their depressive episode are more predictive of suicide attempts than high levels of hopelessness at any point in time.

More recently, Acceptance and Commitment therapy (ACT) has been shown to increase consumer hedonia through mindfulness skills and motivational hedonia through engagement in activities of importance to the individual (172). Ducasse et al. (173) report on a short intervention (seven sessions of ACT) that reduced the frequency and intensity of suicidal ideation in patients with suicidal conduct disorder (a disorder associated with the risk of short-term acting out). Ducasse et al. (83) insist that the crucial problem is not so much whether or not we have experiences that give us pleasure, but rather the relationship that we have with pleasure. Our desires lead us to seek wellbeing, pleasure, and a sense of self-worth in external things. Therefore, when we experience pleasure, we react with a disproportionate attachment or even addiction to the things that are sources of that pleasure. Gordon et al. (174) argue that because of our ever-changing environment, our incessant search for pleasure in external things leads to a greater vulnerability to painful sensations. Therefore, the absence of pleasurable sensation is seen as a situation from which to escape. Suicidal behavior appears to be a faulty coping strategy to escape these unpleasant experiences (83). According to Deci (175), intrinsic motivation is more effective and sustainable for behavioral activation than extrinsic motivation. Focusing on intrinsic motivators allows us to build an “inner compass” that reduces the tendency of our emotions to go with the flow of external events. It is these intrinsic motivators that are targeted in ACT (172) which is effective in treating patients at high risk of suicide by helping them to anchor themselves in a life that is meaningful to them (76).

#### Other psychotherapy strategies

Among psychotherapeutic approaches, mindfulness involves the awareness and acceptance of present moment experiences, whether pleasant or painful (76). Mindfulness has been shown to decrease the perception of physical suffering and improve pain management (176). It has been shown that mindfulness decreases the perception of physical suffering and improves pain management, increases consumption hedonism in patients with chronic pain (177), and reduces the intensity of suicidal ideation (178).

Interpersonal therapies have shown encouraging results in older subjects (179).

Besides the MHEOCC, other suicide prevention plans have been studied and implemented. Michel et al. (180) describe ASSIP (Attempted Suicide Short Intervention Program) as a treatment administered in three 60–90 min sessions within 3 weeks. During the first session, patients tell their personal stories about how they had reached the point of wanting to commit suicide. The interview is video-recorded, with the patients’ written consent. In the second session, the patient and therapist watch selected sequences of the interview, sitting side-by-side. Automatic thoughts, emotions and physiological changes are identified. At the end of the session, the patient is given a homework task (“Suicide is not a rational act”) to be returned, with personal comments, at the next session. At the third session, the patients’ written feedback is discussed. The case conceptualization formulates personal vulnerabilities and suicide triggers, providing the rationale for the need to develop individual warning signs and safety strategies for future suicidal crises. Long-term goals, warning signs, and safety strategies are copied to a credit-card sized folded leaflet and given to the patient, who is instructed to carry this leaflet on them at all times, and to consult it in the event of an emotional crisis. Michel et al. (180) add that ASSIP fulfills the need for an easy to administer, low-cost intervention.

#### Gratitude journal

Usual psychotherapeutic interventions are long and difficult to apply in the context of a suicidal crisis (181). Moreover, they often target unpleasant events from the patient’s past (182). However, the reduction in the intensity of negative emotions is not automatically associated with the appearance of pleasant emotional states (183). Thus, exercises focused on the amplification of positive psychological events seem to be a promising adjunct therapy for suicidal patients (181). Pleasant emotions in patients with a history of suicide attempts have been associated with better problem-solving skills (184). In patients admitted for suicide attempt, low levels of optimism were more predictive of persistent suicidal ideation at discharge than the presence of hopelessness (185). The latter suggested the practice of positive psychology exercises, in particular those centered on gratitude, to improve optimism and decrease despair in patients. Indeed, gratitude is strongly correlated with psychological wellbeing (186) and satisfaction with one’s life (187). It is defined as a positive interpersonal emotion (188) which is experienced in situations where one perceives that one benefits from the positive intentions of others. It has a positive impact on several factors involved in suicidal behavior: stress management and resilience to negative life events (189) through active coping (190), better decision making in complex situations due to a broadening of intentional scope (191) and a sense of belonging (191). Given the shared neurobiological pathways between psychological and physical pain (192), it is interesting to note that positive psychology interventions based on gratitude have an impact in reducing physical pain (193).

A study aimed to evaluate the effectiveness of a short 7-day gratitude diary program in reducing suicidal ideation and psychological pain in a sample of patients admitted for suicidal crisis or suicide attempt (181). The control group wrote a food diary. They found no difference between the groups in the intensity of suicidal ideation (possibly due to the short duration of the intervention). On the other hand, levels of depression and anxiety were significantly improved in the gratitude journal group vs. the control group, which may indicate a partial relief of a state of internal suffering that can be linked to the fact of feeling gratitude. Indeed, gratitude implies appreciating the small everyday things already present in our environment and in our lives, without becoming attached to them. It also involves a reduction in the enjoyment caused by repeated exposure to a positive stimulus (194). There is a hypothesis that gratitude can improve wellbeing directly or indirectly through a buffering effect against depression and negative emotions (195). A study has shown that gratitude is associated with a positive memory bias that increases the salience of positive aspects of daily life and the intensity of pleasant emotions (196). It would thus facilitate access to pleasant memories, leading to hedonic wellbeing. This point is confirmed in a study where, after having compared the gratitude diary vs. a general diary (simple report of the day’s events) for 2 weeks in young women, the authors found that the gratitude diary group had an increase in their hedonic wellbeing and their optimism (197).

### Treatment of the underlying pathology

A certain number of treatments can be effective in the management of suicidal behavior since they have been developed to treat psychiatric illnesses that expose people to the risk of committing suicide. Suicidal behavior and suicide often occur after an acute state of psychiatric illness. When there is an improvement in the symptomatology, the risk of acting out is reduced.

Kessing (198) has shown that the severity of depression is associated with suicidal risk. However, does a patient with depression and suicidal ideation benefit from treatment in the same way as a patient without depression?

To answer this question, Nobile et al. (199) studied two outpatient cohorts of approximately 4,000 depressed patients and compared the evolution of depression (HADS questionnaire) and suicidal ideation (corresponding MADRS item) over 6 weeks after the initiation of antidepressant treatment. In these cohorts, the majority of depressed patients had moderate to severe suicidal ideation at inclusion, 83 and 85.4%, respectively. The risk of a suicide attempt during follow-up was 3 times higher in suicidal patients. Among the subjects with severe suicidal ideation at inclusion, 10% still had severe suicidal ideation at 6 weeks despite the initiation of an antidepressant. Moreover, patients with severe suicidal ideation had a lower rate of remission. This study suggests that “suicidal depression” should be a specific and more severe phenotype of depressive disorder, and highlights the importance of developing effective therapies specific to depression with suicidal ideation, which are risk factors for acting out.

Besides, Fornaro et al. (200) stress that the treatment of suicidal patients with major depressive disorder has been made more difficult by the black box warning for antidepressants required by the FDA in 2004. In 2006, the warning extended to young adults aged up to 25 years, just after media reports appeared about the link between antidepressant and suicide, possibly culminating in an alarmist message. As a consequence, the rates of antidepressant prescriptions declined nearly 50%, especially in children and adolescents. Warnings that antidepressants may increase suicides appear to have backfired as suggested by Isacsson and Allner (201) concluding that “the warning, contrary to its intention, may have increased young suicides by leaving a number of suicidal young persons without treatment with antidepressants.” However, the FDA still seems reluctant to retract the black box warning. Moreover, Fornaro et al. (200) stress that the presence of the black box concerning suicidality has been relayed on several internet websites, with a dangerous exaggeration of the perceived risk. The authors suggest that this black box should be lifted, at least for adult patients, at least until further light would be shed over such an inconclusive and vivid debated matter. Besides, the benefit of antidepressants in reducing the risk associated with depression increases with patient age (202). A study showed the increase of suicidal risk with antidepressants has been associated with younger patients (202). Despite this age-dependent effect, it would be desirable for prescribers to be made aware of this risk for all patients.

### Specific treatments for suicidal crisis?

#### Lithium and clozapine

According to Zalsman et al. (203), lithium and clozapine reduce suicidal risk. Lithium has shown superior efficacy to antidepressants and anticonvulsants (204). Moreover, there are good arguments in favor of the efficacy of lithium not only in ameliorating thymic episodes, but also on suicidal behavior (205). In fact, since the 1970s, a series of both observational and randomized studies have suggested that lithium reduces the risk of suicide (206). In a large observational study of 20,638 patients with bipolar disorder, the rates of suicide and suicide attempts were compared between patients taking lithium and those taking valproate (207). They found a greater anti-suicide effect in the lithium group with the risk of suicide 2.7 times higher (95% confidence interval: 1.1–6.3) in the valproate group. Similar results were found in other observational studies (208–210). Moreover, the effect of lithium on suicide seems to be more important than its effect on mood, because the effectiveness on suicidal behavior was observed even in the absence of mood stabilization (211). This may provide a clinical rationale for continued lithium treatment in patients at risk for suicide who have not responded thymically to lithium (212). On the other hand, there is evidence of an up to 20-fold increase in the risk of suicide when long-term lithium treatment is stopped, particularly if lithium is stopped abruptly (213, 214). This has direct clinical implications, discontinuation of lithium should always be considered with caution, especially in the presence of suicidal risk (215). The benefits of lithium on suicidal behavior could be achieved by a decrease in impulsivity (216, 217) and aggressiveness (215) which are associated with increased suicidal risk (218). Another hypothesis about the mechanism of lithium efficacy is that decision making seems to be improved in patients with bipolar disorder. Indeed, Adida et al. (219) studied euthymic patients with bipolar disorder and showed that patients on lithium and healthy controls were significantly more likely to choose cards from “safe packs” than patients not on lithium. Finally, the regular biological monitoring of patients on lithium required by clinicians could help reduce suicidal behaviors through recognition of early signs of dysphoria, agitation, or suicidal ideation (220).

Clozapine was the first drug approved by the U.S. Food and Drug Administration (FDA) to prevent suicidal behavior (221). A randomized controlled trial demonstrated the superiority of clozapine over olanzapine in reducing the risk of suicidal behavior in patients with schizophrenia or schizoaffective disorder (222). More recently, a meta-analysis has shown that clozapine, when taken regularly, reduces not only the risk of suicide, which confirms the results of another meta-analysis (223), but also other causes of mortality (224). On the other hand, Hennen and Baldessarini (223) question the mechanism of action and efficacy of clozapine in the reduction of suicidal risk. Their questions include:

- What is the pharmacodynamic effect on affective, impulsive, aggressive or other suicide risk factors? (225).
- Does the close medical supervision required specifically for patients on clozapine result in a non-specific protective effect?

#### Ketamine and esketamine

Treatments such as lithium, clozapine, as well as DBT and CBT, have reduced suicide deaths (226–228) and suicide attempt rates (169, 229). Although these treatments and interventions can reduce suicidal risk in the long term, they have not been shown to be effective in the acute setting (230).

Several clinical trials have demonstrated that subanesthetic doses of ketamine have rapid-acting antidepressant (231–234), as well as anti-suicidal properties (235–238), in patients with mood disorders (bipolar and unipolar depression). Given the rapid antidepressant effects of ketamine, this molecule is of great interest in patients at imminent risk of suicide.

Pharmacologically, infusions of ketamine at subanesthetic doses have been associated with reduced suicidal ideation and improved hedonia, independent of depressive symptoms (239, 240). Ketamine therefore has an effect on anhedonia, pain, and psychological pain.

Wilkinson et al. (230) proposed a meta-analysis of 10 studies that showed that a single infusion of ketamine could drastically and rapidly reduce suicidal ideation in depressed patients with an effect maintained at 7 days, regardless of whether suicidal ideation was assessed by the clinician or the patient. More than 50% of patients were free of suicidal ideation within the first 24 h after ketamine injection vs. 20% in the placebo group. However, it still remains to be shown whether ketamine has an effect on suicidal behaviors and acts in the short and medium term, as the literature has so far only reported an effect on suicidal ideation. Moreover, the possibility of a suicidal rebound remains, with possible negative consequences in the weeks or months following exposure to ketamine (241).

Esketamine (S-enantiomer of ketamine) nasal spray was recently approved in the United States and European Union for the indication of resistant depression in adults (Spravato Summary of Product Characteristics 2019; Spravato Prescribing Information 2020). Rapid, robust, and clinically meaningful reduction in depressive symptoms has been demonstrated in resistant depression (242) as well as in depressed patients at imminent risk of suicide (243). This also suggests that esketamine would be an adequate treatment to bridge the delayed onset of efficacy created by the delayed onset of action of conventional antidepressants. Finally, two recent studies show that esketamine nasal spray rapidly reduces depressive symptoms in patients suffering from major depressive episodes with high intentionality to commit suicide (244, 245). Esketamine has shown rapid efficacy in the treatment of resistant depression as well as in the treatment of severe depression associated with suicidal risk (246). The authors add that the effects of esketamine are probably mediated by the GSK3 and BDNF signaling pathways. Interestingly, lithium (a GSK-3 pathway inhibitor), known for its antisuicidal effect, increases the antisuicidal efficacy of esketamine when combined with it.

#### Buprenorphine

Two studies have shown that very low doses of opioid analgesics inhibit separation anxiety in all animal species tested. The authors add that the neuroanatomy of the separation anxiety system overlaps with that of the brain’s “pain matrix” and shares some of its neurotransmitters (247, 248). More generally, social rejection activates the endogenous opioid system in healthy volunteers (249, 250), whereas this activation is impaired in depressed patients (251).

Buprenorphine may have a stronger antidepressant effect through its antagonistic action on kappa receptors (248, 252). It causes much less respiratory depression than other opioids and is therefore safer in the event of overdose (253, 254).

In their study, Yovell et al. (255) aimed to evaluate the efficacy of very low doses of buprenorphine (0.1 mg sublingual 1–2/day) in addition to their background treatment in patients at high risk of suicide without substance use disorders for 4 weeks. In this double-blind randomized controlled trial, they found that these low doses of buprenorphine were associated with a decrease in suicidal ideation. They hypothesize that low-dose buprenorphine is more effective on borderline symptoms (painful feelings of rejection and abandonment) than on anhedonic symptoms.

#### Chronotherapy

**FIGURE 1:**
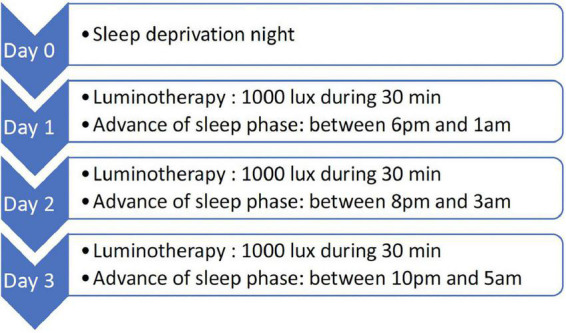
Triple chronotherapy procedure.

Several studies have attributed a rapid antidepressant effect to total sleep deprivation in unipolar and bipolar depression (256–259). However, the clinical usefulness of this technique remained limited because responding patients relapsed rapidly after sleep recovery. The addition of pharmacotherapy (260–263), sleep phase advance (264, 265), and light therapy (260, 265–268) to sleep deprivation, has been shown to be effective in some patients in preventing a depressive relapse. Early studies reported that the combination of total sleep deprivation, sleep phase advance, and light therapy (triple chronotherapy) combined with pharmacological treatment, resulted in rapid improvement of depressive symptoms for 9 weeks (260, 265, 268). But what does the triple chronotherapy procedure involve? First, participants complete the Morningness-Eveningness Questionnaire (MEQ) (269), which predicts the optimal time for light therapy. Sahlem et al. (270) explain it in the following steps (Figure 1).

If the initial encouraging results of triple chronotherapy hold up in further studies, this technique would represent a first choice treatment for hospitalized patients because it is inexpensive, easy to perform, and has minimal side effects (270).

Concerning the clinical utility of triple chronotherapy specifically in patients in suicidal crisis, Sahlem et al. (270) conducted a pilot study with several limitations including a small cohort and no control group. However, they point out that the time in which they observed an effect in their cohort was more rapid than with either pharmacotherapy or psychotherapy. In addition, comparative trials have shown that groups receiving both chronotherapy and pharmacological treatment have a faster and more robust improvement than groups receiving either alone (261, 262, 271, 272).

#### Transcranial direct current stimulation against the “impulsivity” endophenotype

In their study, Gould et al. (103) are interested in animal modeling and propose, not a model of suicide, but rather of a certain number of relatively stable clinical dimensions (“endophenotypes”) which could be a pathway to the development of new therapeutic strategies. If we are able to develop models of impulsivity, aggressiveness, and altered decision-making, then perhaps by acting on different systems we could correct these disturbances in these preclinical models, and then move on to therapeutic strategies in humans.

Impulsivity is understood as a personality dimension as well as a component of the initiation of behaviors (273, 274) that are generally premature, inappropriate, and conceived without foresight or consideration of their consequences (275, 276). From a neurobiological perspective, a strong body of literature suggests that impulsivity, and deficits in impulse control, are associated with neuropsychological, neuroanatomical, and neurotransmitter function abnormalities (277, 278).

Transcranial direct current stimulation (tDCS) is a non-invasive brain stimulation technique that delivers an electrical current through the skull, thereby altering the resting membrane potential of cortical neurons in the targeted area (279–281).

Teti Mayer et al. (282) undertook a review of the literature regarding the effectiveness of tDCS on reducing impulsive behavior in both psychiatric patient populations and healthy volunteers. Of the 92 studies included, 74 studies found improvement in impulsivity-related tasks (45 in the healthy volunteer group, 29 in the psychiatric population). However, these results were often inconsistent. Indeed, the data was fragmented because the studies were based on very small samples with sometimes insufficiently defined criteria (both in terms of population and stimulation parameters).

Another study attempts to evaluate the impact of tDCS on a number of dimensions with the TIMBER program, impaired decision making on the one hand and impulsivity on the other (283). This is a multicentric study that is specifically interested in the reduction of impulsivity in subjects with borderline personality disorder. The interest is to see if this strategy has an impact on different dimensions of impulsivity in order to propose it in the context of care. This program is complemented by a second program (DepImpulse study) whose objective is still to focus on the impulsive dimension, but in a clinical population of patients suffering from recurrent unipolar depression. This program aims to see if it is possible to act not only on the depressive symptomatology, but also specifically on the impulsivity dimensions. This large multicenter study is still in progress as it aims to include a sufficient number of patients.

#### Repetitive transcranial magnetic stimulation

Transcranial magnetic stimulation (TMS) is a non-invasive and painless neuromodulation tool acting on the underlying neuronal excitability. This modulation is achieved by inducing an electrical current discharge in a coil generating a magnetic field, which then induces depolarization of membrane potentials in the cortical tissue beneath the coil, and affects the activity of the associated nerve loop (284). According to Chen et al. (285), high-frequency stimulation (≥ 5 Hz) induces excitatory effects, whereas low-frequency stimulation (≤ 1 Hz) results in inhibitory effects. The efficacy of high-frequency repetitive transcranial magnetic stimulation (rTMS) (repetitive TMS) on the left dorsolateral prefrontal cortex (l-DLPFC) in depression is well established (286). The DLPFC is readily accessible to TMS and is synaptically linked to the limbic system involved in mood regulation (287).

Serafini et al. (284) conducted a systematic review of the literature to assess the association between rTMS and suicidal behavior. The majority of studies identified the l-DLPFC as the preferred area of stimulation. Other areas used were the r-DLPFC (inhibitory stimulus, 1 Hz) and the anterior cingulate cortex. They generally used the standard stimulation protocol (10 Hz) with a number of pulses ranging from 1,200 (288) to 6,000 (289–291). Only one study used DTMS (Deep TMS, administered via an “H1” coil), daily for 4 weeks, for patients with severe drug-resistant depression (292).

DTMS was associated with improvements in behavior and suicidal ideation. Bilateral TMS was significantly more effective than placebo (290, 293), and standard TMS was more effective in combination with antidepressant treatment (284). In the study by Croarkin et al. (294), the reduction in suicidal risk was mediated by an improvement in depressive symptoms. Conversely, Desmyter et al. (295) found that changes in suicidal ideation were independent of an improvement in depression. In the study by Weissman et al. (293) the correlation between depressive symptoms and changes in suicidal ideation was 0.38. Another study found improvements in suicidal ideation, particularly during the first week of treatment (291). Ozcan et al. (296) clearly demonstrated the effectiveness of TMS on suicidal ideation and also noted an improvement in patients’ ability to recognize emotions. A retrospective study based on a sample of 320 depressed patients, treated with a wide variety of TMS protocols, demonstrated a significant improvement in suicidal ideation (292). The same is true in a randomized controlled trial (297). In summary, rTMS has been shown to be effective in alleviating multiple suicidal dimensions including suicidal ideation, intensity of suicidal ideation, suicidal behavior, and suicidal intent (284).

#### Electroconvulsive therapy

Electroconvulsive therapy (ECT) is indicated for patients with a major depressive episode at high risk for suicide (298). Yet, the use of ECT is still debated, in part because there is a lack of data regarding the benefits on suicidal risk (299). As ECT is an effective treatment for depression, particularly in elderly subjects or those with psychotic symptoms (300), it is expected that this treatment will also reduce the risk of suicide. However, although some studies have shown reductions in suicidal ideation (301) and suicidal thoughts (302) associated with electroconvulsive therapy, few studies have examined the association between ECT and suicide rates. There are even contradictory results between studies. Thus, according to a large study (303), patients receiving ECT had an increased risk of suicide compared to those who did not receive ECT. Other smaller studies have found that ECT is associated with either an increased (304) or decreased (305) risk of suicide. Recently, a study examined the association between ECT and suicide risk in patients hospitalized for a major depressive episode. They had a large cohort with 5,525 patients in each group (with and without ECT, three times a week), adjusting for potential confounding factors that might explain the conflicting data from previous studies (306). They found that ECT for depression was associated with a reduced risk of suicide within 3–12 months of hospital care compared with the no ECT group. The association was significant in several analyses, but not in all. Rönnqvist et al. (306) explain their difference in results with Jørgensen et al. (303) by the fact that the latter had included patients with depression of moderate severity, where ECT is not indicated, and even of unspecified severity, as well as outpatients. In contrast, Rönnqvist et al. (306) used much more detailed inclusion factors to minimize confounding. The more severe the depression, the more they found a significant reduction in suicidal risk with ECT. The greatest benefit of ECT was found for psychotic depression, whereas no benefit was noted for moderate depression.

## Discussion

Although suicide in psychiatric hospitals is a rare occurrence, it is a serious event that can be considered a therapeutic failure. In order to improve suicide prevention in hospitals, we have identified scales which have been shown to be effective in accurately assessing the risk of suicide, such as the CAMS and the NSSI-AT. There are also several pharmacological and biological treatments which can reduce suicide risk itself, such as: antidepressants (with particular attention paid to any disinhibiting effect among younger patients); lithium; clozapine; ketamine; esketamine; and ECT. Finally, we recommend the MHEOCC as a dynamic tool which is efficient at securing the hospital environment.

Certain techniques were widely used in the past, but are now outdated, such as anti-suicide contracts.

Other studies suggest two types of suicide prevention techniques that deserve further exploration: (i) Evaluating personal psychological and biological factors potentially linked to suicide risk (anhedonia, psychological pain, expression of gratitude, some endophenotypes, and neural signatures); (ii) therapeutic interventions, such as the use of buprenorphine, non-invasive brain stimulation techniques (rTMS and tDCS) and/or chronotherapy.

In the case of neuroimaging, the way forward is the identification of neural signatures, which could complement and improve the accuracy of clinical assessment of suicide risk.

The unit premises are implicated in the majority of suicides, making them the most important risk factor for intra-hospital suicides. The securing of the environment thus appears to be essential in the prevention of suicide risk. Therefore, the MHEOCC should be generalized to all psychiatric hospitals.

Psychotherapeutic interventions should not be neglected in the management of suicidal patients either. The absence of psychotherapy is an aberration in management. CBT and DBT appear to be the most widely used, and effective, psychotherapies in this indication. Mindfulness, ACT, and gratitude interventions can make a difference in the management of a patient.

Regarding brain stimulation techniques, rTMS has been shown to be effective in alleviating multiple dimensions of suicidality. There is a lack of data concerning the benefits of ECT on suicidal risk. However, the more severe the depression, the more significant the reduction in suicidal risk with ECT.

The implications of the biological and clinical dimensions previously described need to be further explored, in order to confirm the association between them and the suicide risk. We would also need to assess if the intervention on these dimensions can reduce suicide risk. This would allow us to propose specific and personalized pharmacological, psychotherapeutic or brain stimulation treatments for each dimension.

## Author contributions

FC wrote the manuscript and contributed to the conception and design of the study. DJ and NB contributed to the conception and design of the study, as well as the writing of the manuscript. All authors contributed to the article and approved the submitted version.
